# Comparative analysis of the effect of PO administered acid suppressants on gastric pH in healthy cats

**DOI:** 10.1111/jvim.15887

**Published:** 2020-09-04

**Authors:** Phillip Ryan, Adesola Odunayo, Josh Price, Silke Hecht, Shanna Hillsman, Gina Galyon, Joerg Steiner, M. Katherine Tolbert

**Affiliations:** ^1^ Department of Small Animal Clinical Sciences University of Tennessee, College of Veterinary Medicine Knoxville Tennessee USA; ^2^ Department of Small Animal Clinical Sciences Texas A&M University, College of Veterinary Medicine and Biomedical Sciences College Station Texas USA

**Keywords:** bravo monitoring, dexlansoprazole, esomeprazole, feline, lansoprazole, proton pump inhibitor

## Abstract

**Background:**

Proton pump inhibitors (PPIs) are among the most commonly prescribed medications for esophagitis and upper gastrointestinal erosion and ulceration in cats. Newer PPIs such as lansoprazole and esomeprazole are believed to be effective in cats, but the effect of many of these PPIs on gastric pH in cats has not been explored.

**Hypothesis/Objectives:**

To evaluate the efficacy of PO esomeprazole, dexlansoprazole, and lansoprazole on intragastric pH in healthy cats. We hypothesized that esomeprazole and lansoprazole would provide superior acid suppression compared to dexlansoprazole and reach pH goals extrapolated from people for the treatment of esophagitis and duodenal ulceration.

**Animals:**

Twelve healthy research cats.

**Methods:**

Randomized, 3‐way crossover study. Cats were given esomeprazole and lansoprazole at a dosage of 1 mg/kg PO q12h or dexlansoprazole at 6 mg/kg PO q12h. Intragastric pH was recorded at baseline and for 4 days of treatment. Mean pH and the mean percentage time (MPT) intragastric pH was ≥3 or ≥4 were compared among and within treatment groups.

**Results:**

Cats treated with lansoprazole had a lower MPT ± SD of intragastric pH ≥3 (8.8 ± 6.8%) and mean ± SD pH (1.6 ± 0.5) than did cats treated with dexlansoprazole (41.2 ± 34.6% and 3.11 ± 1.6, respectively) or esomeprazole (54 ± 33.8% and 4.1 ± 3.9, respectively;*P* ≤ .04). Esomeprazole was the only treatment that achieved the goals defined for people for the treatment of duodenal ulceration by Day 4 of treatment (MPT ± SD of intragastric pH ≥4 of 77.1 ± 29.2%).

**Conclusions and Clinical Importance:**

Orally administered esomeprazole might be a superior acid suppressant in cats compared to PO lansoprazole or dexlansoprazole.

**Abbreviations**
GI: gastrointestinal
H_2_RA: histamine‐2 receptor antagonist
MPT: mean percentage time
PPI: proton pump inhibitor

## 1 INTRODUCTION

Gastric acid suppressants, including proton pump inhibitors (PPIs) and histamine‐2 receptor antagonists (H_2_RAs), are among the most commonly prescribed medications for esophagitis and upper gastrointestinal (GI) erosion and ulceration in cats. Proton pump inhibitors are superior gastric acid suppressants compared to H_2_RAs and are considered the treatment of choice for upper GI ulceration in cats.^1^, ^2^, ^3^ Indeed, when administered PO to cats, omeprazole, but not famotidine or ranitidine, was the only gastric suppressant when administered q12h that approached treatment goals established for people with duodenal ulcers and gastroesophageal reflux disease.^1^, ^2^ Standardized treatment goals have yet to be established for cats, and therefore, goals established for people have been adopted until more appropriate goals are determined for cats.

Although superior to H_2_RAs that also require q12h administration, PPIs have limitations that can inhibit their effectiveness in cats, including the requirement of administering the medication q12h on an empty stomach. The requirement for q12h administration shortly before meals likely contributes to decreased compliance and increases the potential for treatment failure. Moreover, q12h PO administration has the potential to disrupt the human‐cat bond. More recently developed PPIs, such as the dual delayed‐release PPI, dexlansoprazole, are attractive alternatives because they are intended to have more favorable pharmacokinetic and pharmacodynamic profiles, resulting in longer duration of action and less dependence on the fasting state. However, the effects of these newer PPIs on feline gastric pH in cats are underexplored, and cats might not have a favorable response to dual delayed‐release drugs designed for humans because of the shorter small intestinal length in cats.^4^ Esomeprazole appears to be a superior gastric acid suppressant in dogs^5^, ^6^ and lansoprazole also has been recommended as an effective acid suppressant in cats in conference proceedings and online veterinary forums,^7^, ^8^, ^9^, ^10^, ^11^ but no studies have evaluated the effect of PO esomeprazole or lansoprazole on gastric pH in cats. Therefore, our study objective was to evaluate and compare the efficacy of PO dexlansoprazole, esomeprazole, and lansoprazole in increasing intragastric pH in cats. We hypothesized that esomeprazole and lansoprazole would significantly increase intragastric pH in cats compared to the dual delayed‐release PPI, dexlansoprazole, and reach pH goals for the treatment of esophagitis and duodenal ulceration, as defined for people.

## 2 MATERIALS AND METHODS

### 2.1 Study animals

We studied 12 healthy adult cats from a research colony at the University of Tennessee (9 spayed females and 3 neutered males), aged 3.0 to 5.3 years (median, 5.0 years), and weighing 3.4 to 5.4 kg (median, 4.8 kg). Cats were excluded from the study if they had a history of clinical signs of GI disease including vomiting, inappetence, or diarrhea. Cats also were excluded from the study if abnormalities were present on historical blood tests, if there were any physical examination findings suggestive of systemic or GI disease including poor hair coat, low body or muscle condition score, or abnormalities identified on abdominal palpation or thoracic auscultation. Finally, cats were excluded if they had abnormalities present on baseline blood test results (ie, CBC, serum biochemistry profile, and urinalysis) performed within 12 months of study entry. The number of cats (n = 12) for the study was based on sample size analysis using data from studies comparing the effect of PPIs on gastric pH in animals and humans.^1^, ^12^ Using a conservative correlation of 0.25 and power = 0.8, 10 cats were needed to identify a difference of 20% in mean percentage time (MPT) of gastric pH ≥4 among treatment groups. An additional 2 cats were included to allow for the potential for study dropout. Animals were cared for according to the principles outlined in the National Institutes of Health Guide for the Care and Use of Laboratory Animals (approved Institutional Animal Care and Use Committee protocol for this study #2669 A3).

### 2.2 Study design

In a randomized, open label, 3‐way crossover study design, all cats were given the following drugs PO for 4 consecutive days: (1) dexlansoprazole (Dexlansoprazole; Dexilant delayed‐release capsules; Takeda Pharmaceuticals America, Deerfield, Illinois) at a dosage of 6 mg/kg (range, 5.55‐8.82 mg/kg) q12h; (2) esomeprazole (esomeprazole magnesium capsule, delayed release; BluePoint Laboratories, Cork, Ireland) at a dosage of 1 mg/kg (range, 0.92‐1.47 mg/kg) q12h; and (3) lansoprazole (lansoprazole capsule, delayed release; BluePoint Laboratories) at a dosage of 1 mg/kg (range, 0.92‐1.47 mg/kg) q12h. The dose and frequency of dexlansoprazole was based on standard dosing recommendations for people, as well as a pilot study indicating that dexlansoprazole administered at a dosage of 3 mg/kg q12‐24h was ineffective in increasing intragastric pH in cats. Commercially available capsule formulations of esomeprazole and lansoprazole were not available in appropriate sizes for cats. Accurate dosing for esomeprazole and lansoprazole was achieved by weighing drug granules to the specified dose (5 mg) and filling empty gelatin capsules (size 4, Capsuline, Pompano Beach, Florida). Dexlansoprazole was administered as 30 mg capsules. Cats were randomized to a treatment schedule by a random number generator, so that 4 cats were randomized into each group. Cats were medicated at approximately 6:30 am and pm. To facilitate medicating, cats received 1 teaspoon of canned food (Fancy Feast, Chicken Feast Classic Pate, Nestle Purina PetCare Company, St. Louis, Missouri) q12h, which contained medication on treatment days. Swallowing of the medication was witnessed, and if cats did not voluntarily eat the capsule, it was administered using a pill gun followed by 5 mL of water administered by syringe. The feeding schedule and dosage interval were maintained throughout the study period. Cats received a half cup of dry food (Purina One Tender Selects Blend with Real Salmon Adult Dry Cat Food; Nestle Purina PetCare Company) q12h 30 minutes after being medicated. Cats had unlimited access to water during the pH monitoring period. Clinical signs, including change in attitude, vomiting, and fecal character, were recorded q12h. Litter boxes were evaluated and feces were graded q12h by a veterinary resident (P. Ryan) who scored feces on a score ranging from 1 to 7 based on a standardized fecal scoring system, and diarrhea was defined as a fecal score >4 (Fecal Scoring System; Nestle Purina PetCare Company). Litter boxes were cleaned after grading was performed. An episode of inappetence was defined as consumption of <50% of the meal offered. Vomitus was evaluated for the presence of medication or the pH capsule when it occurred. A period of at least 6 weeks separated treatment groups. After completion of the initial study and review of pH data, a fourth treatment, consisting of 3 cats in which gastric pH responded the least to the capsule formulation of lansoprazole, was added. Lansoprazole was administered to these 3 cats at the previously mentioned dosage and frequency in the form of a PO suspension (lansoprazole, 3 mg/mL, in FIRST‐PPI Suspension Compounding Kit, Cutis Pharma, Wilmington, Massachusetts). The suspension was reconstituted and stored according to the manufacturer's directions. This phase of the study was carried out in a manner identical to the previous 3 treatments in all other respects, but data were not included in statistical analyses.

### 2.3 Intragastric pH monitoring

The Bravo pH monitoring system (Bravo pH capsule with delivery system; Given Imaging, Duluth, Georgia) was placed using radiographic guidance under sedation as previously described.^13^ All pH capsules and receivers were calibrated as previously described according to the manufacturer's instructions. The location of each pH capsule was kept consistent in each cat among treatment groups by utilizing the measurements on the capsule delivery device to measure the distance from the maxillary canine teeth to the area of capsule placement in the gastric fundus based on radiographs. One day before the first treatment period (Day 0, baseline) and after an overnight fast, cats were sedated with 10 μg/kg dexmedetomidine (dexdomitor 0.5 mg/mL injection; Orion Pharma, Espoo, Finland) IV and 0.4 mg/kg butorphanol (torbugesic 10 mg/mL injection; Fort Dodge Animal Health, Fort Dodge, Iowa) IV. The cats were placed in right lateral recumbency. The pH capsule then was blindly introduced transorally into the proximal stomach as previously described.^3^, ^13^ Sedation was reversed with 100 μg/kg atipamezole (Antisedan 5 mg/mL injection; Orion Pharma, Espoo, Finland) IM after pH capsule placement. The pH capsule placement was repeated in the same manner for each treatment.

### 2.4 pH recordings

Intragastric pH recordings were obtained telemetrically at 6‐second sampling intervals. Twenty‐four‐hour intragastric pH recording was initiated immediately after placement and acquired continuously for 120 hours (24‐hour baseline data and treatment Days 1‐4). The corresponding data receivers were kept on the side of each cat's cage during the data acquisition phase. The pH data were uploaded to the computer using a software package provided by the manufacturer (Polygram Net Software; Given Imaging, Yoqneam, Israel) every 24 hours for each monitoring period. The batteries for the receiver were replaced every 48 hours. After data upload, data from the receiver were cleared and the same receiver was used to obtain data for the next 24‐hour period. Data were included from Day 0 and the 4 treatment days for each treatment group. Mean pH and MPT of the intragastric pH ≥ 3 and ≥4 were calculated using the manufacturer's software package (Polygram Net Software; Given Imaging).

### 2.5 Statistical analysis

A 3 treatment, 3 sequence, crossover design with repeated measures was performed to evaluate mean intragastric pH, MPT of intragastric pH ≥3, and MPT of intragastric pH ≥4.^14^, ^15^ Each response measure was analyzed using a repeated measures mixed model analysis of variance (ANOVA) to determine treatment, time (day of treatment), treatment‐by‐time interaction, and carryover effect differences. Unstructured Kronecker product variance/covariance structures were incorporated into each model.^15^ A Shapiro‐Wilk test for normality and QQ plots were used to evaluate normality of ANOVA residuals. Levene's equality of variances test was used to evaluate equality of treatment variances. Box‐and‐whisker plots and studentized residual diagnostics were performed to evaluate each mixed model for the presence of outliers. All statistical assumptions regarding normality and equality of variances were met after a log transformation was applied to each response measure. Statistical analysis was performed using commercial software (SAS software, version 9.4, Cary, North Carolina, Release TS1M6).^16^ Statistical significance was defined as *P* ≤ .05.

## 3 RESULTS

### 3.1 pH capsule placement

All pH capsules remained in place for the entirety of the 120‐hour study period. For 2 cats in each of the treatment groups, data for treatment Day 4 was inadvertently not recorded and therefore this data was not included in the Day 4 analyses.

### 3.2 Intragastric pH recording

**FIGURE 1:**
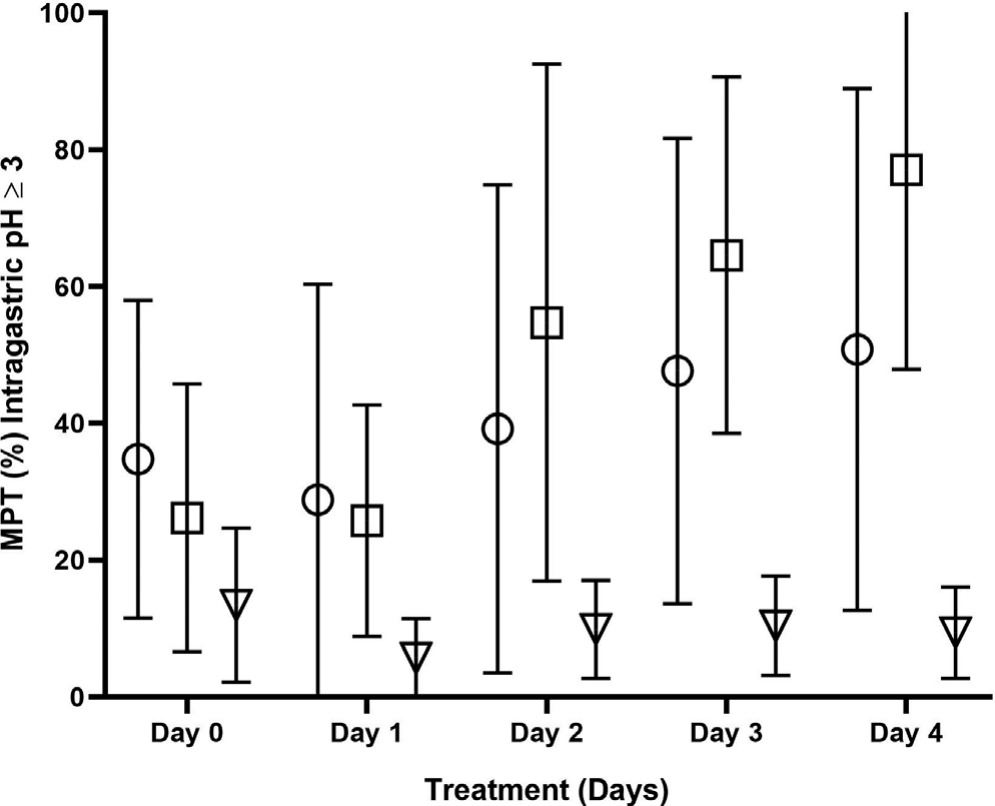
The mean ± SD percentage time (MPT, %) of intragastric pH ≥3 for all cats administered 6 mg/kg q12 h dexlansoprazole (circles), 1 mg/kg q12h esomeprazole (squares), or 1 mg/kg q12h lansoprazole (triangles) PO on treatment Days 1 to 4. Significant differences were found among treatments (*P* = .04) and over time (*P* = .002), but not treatment‐by‐time (*P* = .24). The MPT of intragastric pH ≥3 was significantly lower on Day 1 compared to all other days regardless of the treatment received (*P* ≤ .02, for all). Post hoc tests revealed that, on average, cats treated with lansoprazole had a lower MPT of intragastric pH ≥3 than cats treated with dexlansoprazole or esomeprazole (*P* = .03, for each). No significant differences were observed between dexlansoprazole and esomeprazole, Data presented from 12 cats except where indicated by *, where n = 10 cats

**FIGURE 2:**
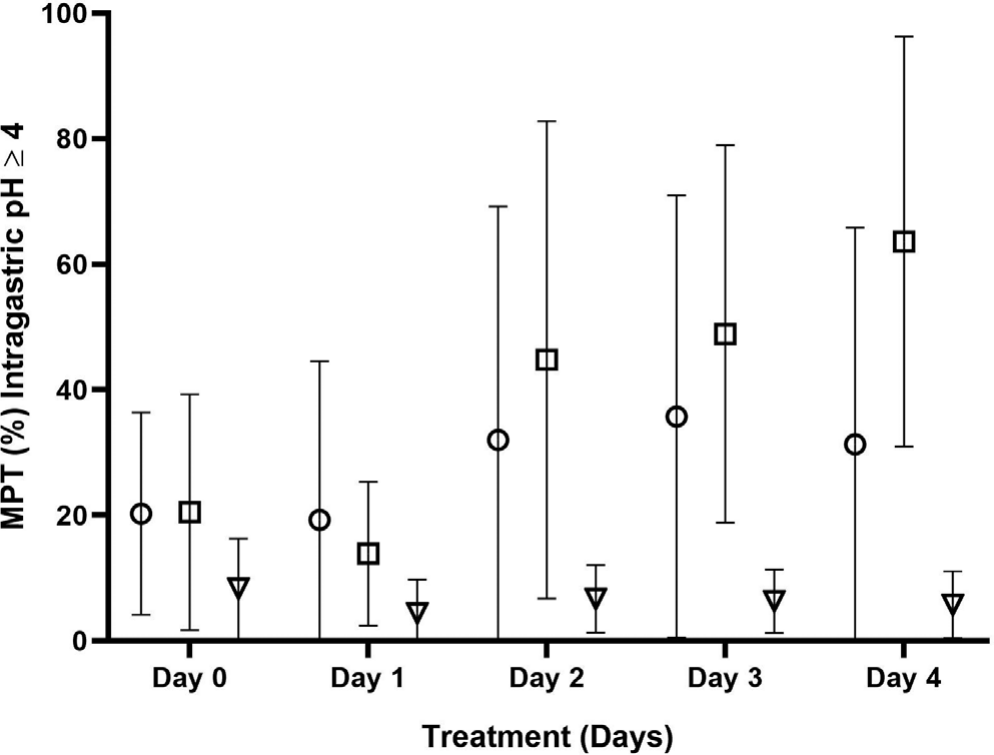
The mean ± SD percentage time (MPT, %) ofintragastric pH ≥4 for all cats administered 6 mg/kg q12h dexlansoprazole (circles), 1 mg/kg q12h esomeprazole (squares), or 1 mg/kg q12h lansoprazole (triangles) PO on treatment Days 1 to 4. Significant differences were observed over time (*P* = .01) but not by treatment (*P* = .08) or treatment‐by‐time (*P* = .14). Post hoc tests revealed that, on average, MPT of intragastric pH ≥4 was significantly lower on Day 1 compared to Days 2 through 4 (*P* ≤ .005, for all). Data presented from 12 cats except where indicated by *, where n = 10 cats

**FIGURE 3:**
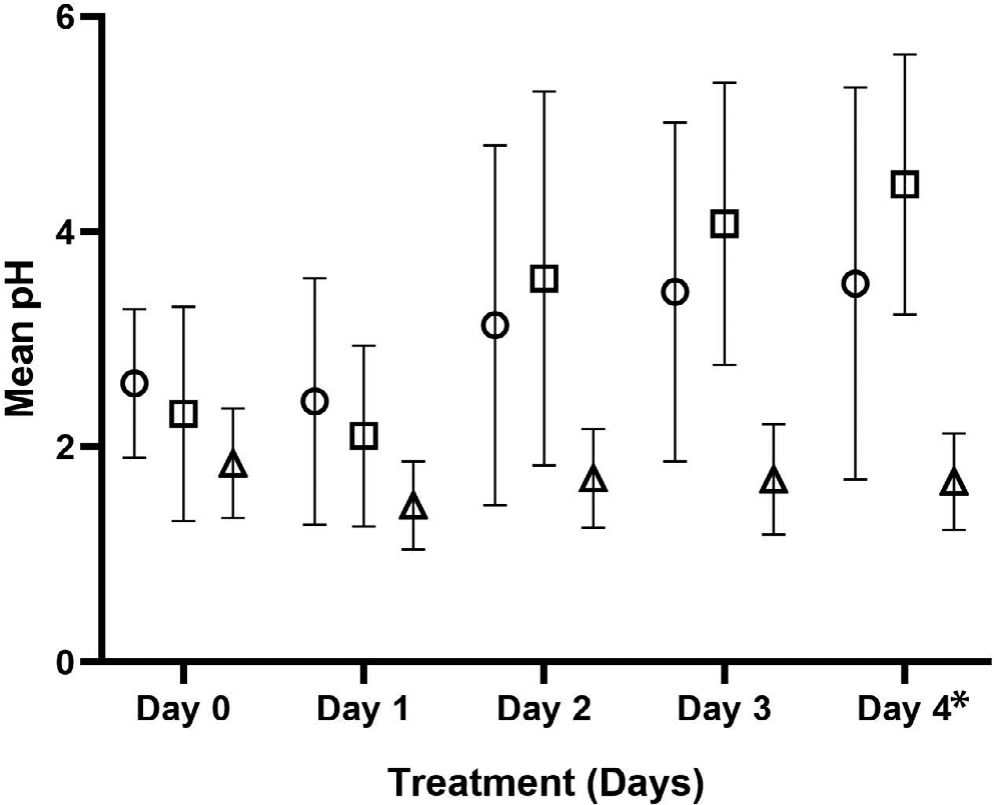
The mean ± SD intragastric pH for all cats administered 6 mg/kg q12h dexlansoprazole (circles), 1 mg/kg q12h esomeprazole (squares), or 1 mg/kg q12h lansoprazole (triangles) PO on treatment Days 1 to 4. Significant differences in mean pH were observed over time based on the treatment received (*P* = .03). On Days 0 and 1, no differences in mean pH were observed among treatments, however, the mean gastric pH for both dexlansoprazole and esomeprazole were significantly increased when compared to lansoprazole (*P* ≤ .02 for each) on Days 2 to 4. Dexlansoprazole and esomeprazole did not differ from each other on any days. For esomeprazole, Day 0 and 1 did not differ, but the mean pH significantly increased over time for Days 2‐4 compared to Day 1 (*P* ≤ .005, for all). The mean pH did not significantly differ over time for dexlansoprazole. For lansoprazole, a significant decrease in mean pH was observed between Days 0 and 1 (*P* = .003) and increase between Days 1 and 2 (*P* = .04), but no other significant differences were observed. Data presented from 12 cats except where indicated by *, where n = 10 cats

Mean intragastric pH and MPT of intragastric pH ≥3 and 4 are depicted in Figures 1, 2, 3, respectively. Significant differences in MPT of intragastric pH ≥3 (Figure 1) were found between treatments (*P* = .04) and over time (*P* = .002), but not treatment‐by‐time (*P* = .24). Post hoc tests determined that MPT of intragastric pH ≥3 was significantly lower on Day 1 compared to all other days, regardless of treatment received (*P* ≤ .02, for all). Cats treated with lansoprazole had a lower MPT of intragastric pH ≥3 than did cats treated with dexlansoprazole or esomeprazole (*P* = .03, for each). No significant differences were observed between dexlansoprazole and esomeprazole.

Significant differences in MPT of intragastric pH ≥4 were observed over time (*P* = .01), but not by treatment (*P* = .08) or treatment‐by‐time (*P* = .14). Post hoc tests indicated that, on average, MPT of intragastric pH ≥4 was significantly lower on Day 1 compared to Days 2 through 4 (*P* ≤ .005, for all). The MPT of intragastric pH ≥4 on Day 0 did not differ significantly from that of Day 1 for any treatment. No differences were observed on Days 2 to 4 for any treatment.

Significant differences in mean intragastric pH were observed over time based on the treatment received (*P* = .03). On Days 0 and 1, no differences in mean pH were observed among treatments, but on Days 2 to 4 mean gastric pH for both dexlansoprazole and esomeprazole was significantly increased when compared to that of lansoprazole (*P* ≤ .02 for each). Mean intragastric pH for dexlansoprazole and esomeprazole did not differ from each other on any of the treatment days. For esomeprazole, mean intragastric pH on Days 0 and 1 did not differ, but mean pH significantly increased over time for Days 2 to 4 compared to Day 1 (*P* ≤ .005, for all). In contrast, mean intragastric pH did not significantly differ over time for dexlansoprazole. For lansoprazole, a significant decrease in mean intragastric pH was observed between Days 0 and 1 (*P* = .003) and an increase between Days 1 and 2 (*P* = .04), but no other significant differences were observed. No significant differences were found among periods or among treatments on Day 0 for any pH response measure. Thus, no significant carryover effects were found, indicating the washout period between treatments was adequate.

### 3.3 Effects of lansoprazole oral liquid formulation

Similar to what was described for the lansoprazole capsule formulation, the liquid lansoprazole suspension had no noticeable effect on increasing intragastric pH in the 3 cats over time (Supplementary Figure 1).

### 3.4 Adverse events

None of the cats were excluded from the study. All treatments were generally well tolerated. There were no episodes for which cats consumed <50% of the food offered. The total number of vomiting episodes for treatments was 7 (4 and 2 episodes in 2 cats each receiving esomeprazole and 1 episode for 1 cat receiving dexlansoprazole). None of these vomiting episodes occurred immediately after medicating the cats. The mean ± SD fecal scores for cats treated with dexlansoprazole, esomeprazole, or lansoprazole for all treatment days were 1.9 ± 0.4, 2.1 ± 0.7, and 2.0 ± 0.3, respectively. One cat with 4 vomiting episodes also had a fecal score of 6 on treatment Days 3 and 4 when treated with esomeprazole and was mildly lethargic but had no changes in appetite.

## 4 DISCUSSION

The optimal degree of acid suppression for the treatment of gastroduodenal ulceration or esophagitis in cats is unknown. Therefore, treatment goals for people with duodenal ulceration^17^ and esophagitis^18^ were used for purposes of comparing different PPIs in our study. In previous studies, although PO omeprazole proved to be superior in increasing gastric pH in cats compared to the H_2_RAs, famotidine and ranitidine, omeprazole still failed to achieve the aforementioned goals.^1^, ^2^ This failure prompted us to explore the effect of the other commercially available PO PPIs, esomeprazole, dexlansoprazole, and lansoprazole, on increasing the gastric pH of cats. Of these, esomeprazole was the only treatment that led to a statistically significant increase in mean intragastric pH over time. Moreover, esomeprazole was the only treatment that achieved the goal for the treatment of duodenal ulceration with a MPT ± SD of intragastric pH >3 of 77 ± 29% by treatment Day 4. Day 4 is the day by which all drugs were assumed to have reached steady state for inhibition of gastric acid secretion, although in our study no significant differences were found for Days 2 to 4 for any treatment. This result is slightly higher than that previously reported^2^ for q12h PO omeprazole capsules (67.0 ± 24.0% averaged over Days 4‐7). Omeprazole is a racemic mixture of the 2 enantiomers, R‐omeprazole and S‐omeprazole, whereas esomeprazole contains only the S‐isomer of omeprazole. The S‐omeprazole is less sensitive to metabolism by Cytochrome P450 2C19 (CYP2C19) compared to R‐omeprazole, and maintains better drug plasma concentrations in people.^19^ However, the role of CYP2C in xenobiotic metabolism in cats recently has been questioned because researchers identified negligible to no functional CYP2C hepatic protein in cats and suggested that CYP2C does not play a major role in the systemic clearance of any xenobiotics in cat.^20^ Therefore, additional comparative pharmacodynamic studies in the same cohort of cats are needed before definitive conclusions can be drawn regarding the superiority of esomeprazole over omeprazole in cats.

Dexlansoprazole, the R‐enantiomer of lansoprazole, was not significantly different from esomeprazole in MPT of intragastric pH ≥3 or 4 and was superior to lansoprazole in increasing gastric pH, but, unlike esomeprazole, failed to reach any pH goals used in humans, despite being administered at 6 times the dosage of esomeprazole and dexlansoprazole. Further study is warranted to determine if higher dosages would be more effective at increasing gastric pH in cats. However, the daily cost of dexlansoprazole (30 mg) per cat at the time of study was $19 because it is currently only available in brand name form. Dexlansoprazole releases active drug in the proximal duodenum at a pH 5.0 and later in the distal portion of the small intestine at a pH of 7.0, providing sustained acid suppression in people. A single dose of dexlansoprazole has been reported to be superior to esomeprazole in increasing gastric pH and improves nighttime pH control in healthy adults.^21^ In an ongoing study,^22^ we determined that the pH of the upper small intestine of healthy cats may be higher than that reported for people and dogs, which would result in an immediate release of all drug rather than a sustained, slower release as designed for people. This might explain the decreased efficacy we documented in cats as compared to that reported for people.

Orally administered lansoprazole capsules, on the other hand, had no detectable effect on increasing gastric pH on any treatment day compared to no treatment (Day 0). This finding was surprising, given that lansoprazole is comparable to omeprazole in healing acid‐related injury in people. Furthermore, IV lansoprazole was similar to omeprazole in cats in the inhibition of acid secretion in a gastric fistula model study.^23^ The reasons for the lack of effect of PO lansoprazole in our study are unclear. Esomeprazole and lansoprazole granules were packaged in the same gelatin capsules, ruling out capsule failure as the underlying cause. Delayed gastric emptying or faster intestinal transit as a reason for a lack of effect also was considered unlikely given the crossover design. Both drugs are dependent on the CYP3A and CYP2C19 enzymes for metabolism and were administered to all cats, making it unlikely that metabolism disparity was a predominant contributing factor. Differences in enteric coating of the capsule granules, or other drug layering characteristics, were considered because esomeprazole was coated with methacrylic acid, dexlansoprazole was coated with methacrylate‐methacrylic acid and methacrylic acid‐ethyl acrylate copolymers, and lansoprazole was coated with methacrylic acid‐ethyl acrylate copolymer (1:1) type A. To explore this potential explanation, we undertook a small pilot study to evaluate the effect of lansoprazole suspension and determined that it too failed to increase the gastric pH in the 3 cats tested. The reasons for failure of PO lansoprazole in our study cats remain unclear. Our results highlight the need for veterinarians and pharmaceutical companies to explore and design drugs such as PPIs specifically for use in cats.

We identified a significant decrease in mean intragastric pH between Days 0 and 1 when cats received lansoprazole. We believe this observation is a result of an increase in intragastric pH on Day 0 secondary to biliary reflux after sedation. This phenomenon often is less evident with potent acid suppressants because they have a greater effect on gastric pH on Day 1, but can be observed with placebo or less potent acid suppressant as was the case with lansoprazole.

Pharmacodynamic studies can be complicated by many physiologic variables, including genetic and metabolism differences among individuals, disease states, environmental disparities, aging, and the presence of other drugs. We chose to limit these variables by studying the effects of 3 different PPIs in healthy, genetically similar cats fed the same diet, living in the same environment, and being free of disease. Our results serve as the background for future studies in cats with erosive and ulcerative upper GI disease. Based on the current, as well as historical studies, we conclude that esomeprazole might be a superior acid suppressant in cats and warrants further study.

## CONFLICT OF INTEREST DECLARATION

Authors declare no conflict of interest.

## OFF‐LABEL ANTIMICROBIAL DECLARATION

Authors declare no off‐label use of antimicrobials.

## INSTITUTIONAL ANIMAL CARE AND USE COMMITTEE (IACUC) OR OTHER APPROVAL DECLARATION

The IACUC at The University of Tennessee approved the protocol for this study (Approval #2634‐0718).

## HUMAN ETHICS APPROVAL DECLARATION

Authors declare human ethics approval was not needed for this study.

## Supporting information


**Supplementary Figure 1** The mean percentage time (MPT, %) intragastric pH ≥3 (A), ≥ 4 (B), and mean intragastric pH (C) for all cats administered 1 mg/kg q12hr esomeprazole, 1 mg/kg q12hr lansoprazole capsules or suspension, or 6 mg/kg q12hr dexlansoprazole orally on treatment days 1‐4. Horizontal and vertical lines represent the means and standard deviations, respectively, for each day. Individual cat data are represented by open circles (dexlansoprazole), squares (esomeprazole), triangles (lansoprazole capsules), and closed circles (lansoprazole suspension).
