# Supplementary figures and images for: Comparative analysis of the effect of PO administered acid suppressants on gastric pH in healthy cats
Source: J Vet Intern Med. 2020 Sep 4;34(5):1879–85. doi: 10.1111/jvim.15887 (PMC7517516; doi:10.1111/jvim.15887)

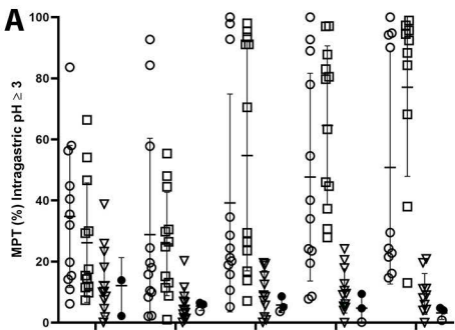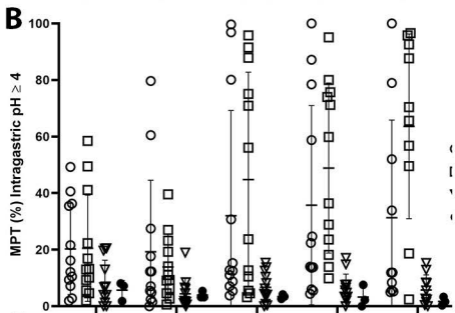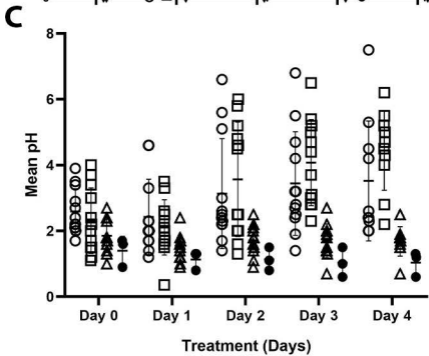

Supplement: Supplementary file 1 — Supplementary Figure 1 The mean percentage time (MPT, %) intragastric pH ≥3 (A), ≥ 4 (B), and mean intragastric pH (C) for all cats administered 1 mg/kg q12hr esomeprazole, 1 mg/kg q12hr lansoprazole capsules or suspension, or 6 mg/kg q12hr dexlansoprazole orally on treatment days 1‐4. Horizontal and vertical lines represent the means and standard deviations, respectively, for each day. Individual cat data are represented by open circles (dexlansoprazole), squares (esomeprazole), triangles (lansoprazole capsules), and closed circles (lansoprazole suspension). [file JVIM-34-1879-s001.pdf]
